# Supplementary material for: Comparative multiomics analysis of cell physiological state after culture in a basket bioreactor
Source: Sci Rep. 2022 Nov 23;12:20161. doi: 10.1038/s41598-022-24687-4 (PMC9686226; doi:10.1038/s41598-022-24687-4)
Supplement: Supplementary file 1 — Supplementary Information 1. [file 41598_2022_24687_MOESM1_ESM.zip › raw data/Proteomic raw data/6.KEGGenrichment/EG.vs.CG/DOWN/pathway/src/00310.html]

map00310


- K06101, K11427, K11420, K09186, K09187, K09188, K14959, K09189, K15588, K11424, K11425, K11422, K11423, K19199, K11431, K11428, K11421, K18494, K11433, K11419, K11429, K17451, K11430, K11432, K20795, K20796, 2.1.1.43, R03875"

- Down regulated proteins


A0A0D9R3X6(Description:Histone-lysine N-methyltransferase EZH2;Gene:EZH2;log2FC:-2.0882)

- K06101, K11427, K11420, K09186, K09187, K09188, K14959, K09189, K15588, K11424, K11425, K11422, K11423, K19199, K11431, K11428, K11421, K18494, K11433, K11419, K11429, K17451, K11430, K11432, K20795, K20796, 2.1.1.43, R04866"

- Down regulated proteins


A0A0D9R3X6(Description:Histone-lysine N-methyltransferase EZH2;Gene:EZH2;log2FC:-2.0882)

- K06101, K11427, K11420, K09186, K09187, K09188, K14959, K09189, K15588, K11424, K11425, K11422, K11423, K19199, K11431, K11428, K11421, K18494, K11433, K11419, K11429, K17451, K11430, K11432, K20795, K20796, 2.1.1.43, R04867"

- Down regulated proteins


A0A0D9R3X6(Description:Histone-lysine N-methyltransferase EZH2;Gene:EZH2;log2FC:-2.0882)

Close
